# 

**Published:** 1953

**Authors:** 


### Contents

| | | Page |
|---|---|---|
| FOREWORD | *Sir Philip Morris, C.B.E., M.A., LL.D.* | 1 |
| "MARASMUS" | *Prof. A. V. Neale* | 3 |
| UNDERWATER BREATHING | *Dr. Ronald Woolmer* | 9 |
| MEDICINE AT HOME AND ABROAD | *Dr. John Apley* | 16 |
| THE ELDERLY CHRONIC SICK | | 20 |
| ANNOTATION | | 20 |
| GOOD SENSE ABOUT ANTIBIOTICS | | |
| NEWS FROM SOCIETIES | | 20 |
| BRISTOL MEDICO-CHIRURGICAL SOCIETY | | |
| CLINICAL SOCIETY OF BATH | | |
| PLYMOUTH MEDICAL SOCIETY | | |
| DEVON AND EXETER MEDICO-CHIRURGICAL SOCIETY | | |
| SOUTH SOMERSET MEDICAL CLUB | | |
| THE SOUTH-WEST REGIONAL PAEDIATRIC CLUB | | |
| TORQUAY AND DISTRICT MEDICAL SOCIETY | | |
| APPOINTMENTS | | 23 |
| NOTES AND NEWS | | 27 |
| CORONATION HONOURS | | |
| SCHOLARSHIPS AND PRIZES | | |
| HIGHER DEGREES AND DIPLOMAS | | |
| UNIVERSITY OF BRISTOL | | |
| WHERE CAN I LOOK IT UP? | | |
| BRISTOL MARRIAGE AND FAMILY GUIDANCE COUNCIL | | |

